# Perceived Quality of In-Service Communication and Counseling Among Adolescents Undergoing Voluntary Medical Male Circumcision

**DOI:** 10.1093/cid/cix971

**Published:** 2018-04-03

**Authors:** Lynn M Van Lith, Elizabeth C Mallalieu, Eshan U Patel, Kim H Dam, Michelle R Kaufman, Karin Hatzold, Arik V Marcell, Webster Mavhu, Catherine Kahabuka, Lusanda Mahlasela, Emmanuel Njeuhmeli, Kim Seifert Ahanda, Getrude Ncube, Gissenge Lija, Collen Bonnecwe, Aaron A R Tobian

**Affiliations:** 1 Johns Hopkins Center for Communication Programs, Baltimore, Maryland; 2 Department of Pathology, Johns Hopkins University School of Medicine, Baltimore, Maryland; 3 Department of Health, Behavior and Society, Johns Hopkins University Bloomberg School of Public Health, Baltimore, Maryland; 4 Population Services International, Harare, Zimbabwe; 5 Department of Pediatrics, Johns Hopkins University School of Medicine, Baltimore, Maryland; 6 Centre for Sexual Health and HIV/AIDS Research, Harare, Zimbabwe; 7 CSK Research Solutions, Dar es Salaam, Tanzania; 8 Centre for Communication Impact, Pretoria, South Africa; 9 Office of HIV/AIDS, Global Health Bureau, United States Agency for International Development, Washington, District of Columbia; 10 Ministry of Health and Child Care, Harare, Zimbabwe; 11 Ministry of Health, Community Development, Gender, Elderly and Children, Dar es Salaam, Tanzania; 12 National Department of Health, Pretoria, South Africa

**Keywords:** adolescents, voluntary medical male circumcision, counseling, in-service communication, sub-Saharan Africa

## Abstract

**Background:**

Experience with providers shapes the quality of adolescent health services, including voluntary medical male circumcision (VMMC). This study examined the perceived quality of in-service communication and counseling during adolescent VMMC services.

**Methods:**

A postprocedure quantitative survey measuring overall satisfaction, comfort, perceived quality of in-service communication and counseling, and perceived quality of facility-level factors was administered across 14 VMMC sites in South Africa, Tanzania, and Zimbabwe. Participants were adolescent male clients aged 10–14 years (n = 836) and 15–19 years (n = 457) and completed the survey 7 to 10 days following VMMC. Adjusted prevalence ratios (aPRs) were estimated by multivariable modified Poisson regression with generalized estimating equations and robust variance estimation to account for site-level clustering.

**Results:**

Of 10- to 14-year-olds and 15- to 19-year-olds, 97.7% and 98.7%, respectively, reported they were either satisfied or very satisfied with their VMMC counseling experience. Most were also very likely or somewhat likely (93.6% of 10- to 14-year olds and 94.7% of 15- to 19-year olds) to recommend VMMC to their peers. On a 9-point scale, the median perceived quality of in-service (counselor) communication was 9 (interquartile range [IQR], 8–9) among 15- to 19-year-olds and 8 (IQR, 7–9) among 10- to 14-year-olds. The 10- to 14-year-olds were more likely than 15- to 19-year-olds to perceive a lower quality of in-service (counselor) communication (score <7; 21.5% vs. 8.2%; aPR, 1.61 [95% confidence interval, 1.33–1.95]). Most adolescents were more comfortable with a male rather than female counselor and provider. Adolescents of all ages wanted more discussion about pain, wound care, and healing time.

**Conclusions:**

Adolescents perceive the quality of in-service communication as high and recommend VMMC to their peers; however, many adolescents desire more discussion about key topics outlined in World Health Organization guidance.

Adolescents’ experiences with a healthcare provider can shape the perceived quality of adolescent sexual and reproductive health (ASRH) services. A systematic literature review focused on ASRH services in sub-Saharan Africa highlights evidence of a disregard for privacy, judgmental attitudes toward adolescents seeking services, and a lack of respect, all of which negatively affect adolescent–provider interaction [1]. Insufficient training and inadequate guidelines outlining how best to address adolescents’ sexual and reproductive health needs are barriers for providers, with the result that the counseling needs of young people remain largely ignored [2].

Global guidance on the standards for quality health services for adolescents, as set forth by the World Health Organization (WHO), advocates for competency-based trainings in adolescent health, age-appropriate tools and materials, and ongoing monitoring of the quality of health education and counseling by providers [3, 4]. This includes assessments of the adolescents’ experience of care regarding confidentiality, privacy, and friendly and nonjudgmental provider attitudes. The core competencies in adolescent health for providers, also articulated by WHO, highlight the need to approach every adolescent as an individual with varying needs, levels of maturity, and degrees of health literacy, while also understanding how the stages of adolescent development (physical, cognitive, etc) influence an adolescent’s behavior [5, 6].

Adolescent experiences during voluntary medical male circumcision (VMMC), which provides one of the few entry points through which health services can reach male adolescents, have the potential to influence future health-seeking behavior. VMMC services constitute an important opportunity to contribute to structural, policy, and healthcare setting changes aimed at improving the health and well-being of adolescent males [7]. Research shows that when boys feel they are in a safe and confidential space, including with a healthcare provider, they desire a genuine and caring relationship and will talk honestly about their experiences [3, 8]. Adolescents, regardless of country and socio-economic level, value respectful patient-centered care, appropriate provision of information, and high-quality communication, attributes which are already endorsed by the WHO [9].

The WHO/Joint United Nations Programme on HIV/AIDS Framework for VMMC 2021 includes targets for reaching 90% of males 10–29 years of age by 2021 with VMMC services that include “age-appropriate, comprehensive sexuality and health education” for 10- to 14-year-olds and “detailed sexual health counseling” among other components for 15- to 19-year-olds [10]. Given that, to date, the majority of individuals seeking VMMC across most priority countries are adolescents. This study sought to better understand male adolescents’ perceptions of the quality of in-service communication and counseling they experienced while receiving VMMC services.

## METHODS

### Ethics Statement

The Tanzania National Institute for Medical Research, the Human Sciences Research Council in South Africa, the Medical Research Council of Zimbabwe, and the Johns Hopkins Bloomberg School of Public Health Institutional Review Board all approved the study prior to data collection. Parent permission was obtained for adolescent males <18 years of age, and assent/consent was obtained for all adolescents.

### Study Setting and Design

Participants were recruited from 14 facilities offering VMMC services to adolescents in South Africa, Tanzania, and Zimbabwe [11]. Data were collected from June to September 2015 in Tanzania, August to December 2015 in Zimbabwe, and February to June 2016 in South Africa. A preprocedure quantitative survey was conducted with a convenience sample of adolescent males aged 10–19 years seeking VMMC (n = 1526) [11]. Participants completed a postprocedure quantitative survey 7–10 days following VMMC (n = 1293). There was a 15.3% loss-to-follow up rate; associations with loss to follow up have been described elsewhere [12]. The present study was limited to 1293 participants who completed the postprocedure survey (South Africa, n = 299; Tanzania, n = 498; Zimbabwe, n = 496).

### Measures

#### Satisfaction With Voluntary Medical Male Circumcision Experience

Overall satisfaction with the VMMC experience and likelihood of recommending VMMC to male peers were measured using ordinal 4-point Likert response scales (very dissatisfied, dissatisfied, satisfied, very satisfied; not at all likely, a little likely, somewhat likely, very likely).

#### Comfort With Gender of Counselor/Providers

Adolescents’ gender preference for providers was measured with a 3-point Likert response scale (less comfortable, did not change comfort, more comfortable). Participants were asked about their level of comfort with having a male or female counselor during the individual and group preprocedure counseling sessions. In addition, participants were asked about their level of comfort with having a male or female provider perform the VMMC or check the wound during follow-up.

#### Perceived Quality of In-Service (Counselor) Communication

A scale to measure the perceived quality of in-service (counselor) communication, which taps into the quality of patient–counselor interpersonal interactions, was developed using 9 items adapted from the WHO Quality Assessment Guidelines [4] and the GATHER Tool [13]. Binary responses were coded as 0 (“no/don’t remember”) or 1 (“yes”). Exploratory factor analysis of all 9 items was conducted using a tetrachoric correlation matrix (Supplementary Table 1). Principal components analysis, scree plot visualization, and parallel analysis (1000 repetitions) suggested extraction of a single factor that explained 57.6% of the variance. Factor loadings (range, 0.55–0.84) confirmed the decision to retain all items in the scale. The composite scale had good internal consistency (KR-20 = 0.74) and had a theoretical range between 0 and 9, where a lower score indicated poorer perceived quality of in-service communication.

#### Perceived Quality of Facility-Level Factors in Service Delivery

Participants were asked 7 items related to facility-level factors that could influence the perceived quality of their VMMC experience (eg, “Were the working days and hours of the facility convenient for you?”). Responses were coded as 0 (“no/don’t remember”) or 1 (“yes”).

#### Perceptions and Preferences in Counseling Content

To explore participants’ perceptions of the content of counseling, they were asked (1) “What do you wish had been discussed (more) during counseling? and (2) “What do you wish had not been discussed during counseling?” Participants could provide multiple unprompted responses; interviewers had a predetermined list of relevant categories but detailed responses could also be documented. It should be noted that due to the open-ended nature of this question, interviewers did not consistently administer this question to all adolescents. Furthermore, it was not clearly coded if the interviewer skipped the question or the adolescent responded “nothing” to each question. Thus, these findings should be cautiously interpreted.

### Data Analysis

Differences in the distribution of ordinal Likert responses between age-groups (10–14 vs 15–19 years) were assessed using the nonparametric Somers D test with Fisher Z transformation; the Somers D tests accounted for facility-level clustering using the delta-jackknife method and adjusted for country by stratification [14, 15].

Correlates of a perceived quality of counselor communication score <7/9 (ie, the proportion of participants who perceived the poorest quality of counselor-adolescent communication) were examined. This threshold was selected based on the distribution of scores among the entire sample population (median, 8; interquartile range [IQR], 7–9). For this analysis, modified Poisson regression models were used with generalized estimating equations and robust variance estimation to account for facility-level clustering. Factors shown to have an association with the outcome after adjustment for country were included in the final multivariable model (*P* < .15), with the exception of age group and receipt of a postprocedure counseling session, which were included regardless of statistical significance. Only some participants experienced both a preprocedure and postprocedure counseling session, and since the outcome variable did not differentiate between the two, a sensitivity analysis limited to participants who self-reported only receiving preprocedure counseling was conducted. All analyses were among complete cases only.

Analyses were performed using Stata SE software version 14.2 (StataCorp, College Station, Texas).

## RESULTS

### Study Population

A total of 1293 adolescents completed the preprocedure and postprocedure survey, including 836 (64.7%) 10- to 14-year-olds and 457 (35.3%) 15- to 19-year-olds across South Africa, Tanzania, and Zimbabwe. Table 1 depicts the age breakdown by country, indicating their VMMC facility setting as urban (53.8%), periurban (14.8%), or rural (31.3%). Of the participants who participated in the survey, 48.8% of 10- to 14-year-olds and 19.0% of 15- to 19-year-olds received both individual and group counseling prior to receiving services. Of remaining participants, 16.6% of 10- to 14-year-olds and 42.2% of 15- to 19-year-olds received only individual counseling, and 33.7% and 38.3%, respectively, received only group counseling. Although all respondents reported receiving either individual or group preprocedure counseling, more than three-quarters (78.5%) of the 10- to 14-year-olds did not receive a postprocedure counseling session, nor did half (51.0%) of 15- to 19-year-olds. Just over half (56.1%) of parents attended the preprocedure counseling session with 10- to 14-year-olds; 12.5% attended for the older age group.

**Table 1.** Characteristics of the Study Population

Characteristic	Age 10–14 y (n = 836)	Age 15–19 y (n = 457)	Overall (N = 1293)
Country			
South Africa	187 (22.4)	112 (24.5)	299 (23.1)
Tanzania	413 (49.4)	85 (18.6)	498 (38.5)
Zimbabwe	236 (28.2)	260 (56.9)	496 (38.4)
Facility setting			
Urban	429 (51.3)	267 (58.4)	696 (53.8)
Periurban	149 (17.8)	43 (9.4)	192 (14.8)
Rural	258 (30.9)	147 (32.2)	405 (31.3)
Preprocedure counseling			
Individual only	139 (16.6)	193 (42.2)	332 (25.7)
Group only	282 (33.7)	175 (38.3)	457 (35.3)
Both	408 (48.8)	87 (19.0)	495 (38.3)
Parent/guardian attendance at preprocedure counseling session			
No	361 (43.2)	399 (87.3)	760 (58.8)
Yes	469 (56.1)	57 (12.5)	526 (40.7)
Received a postprocedure counseling session			
No	656 (78.5)	233 (51.0)	889 (68.8)
Yes	180 (21.5)	224 (49.0)	404 (31.2)
Preprocedure counselor gender			
Male only	213 (25.5)	191 (41.8)	404 (31.2)
Female only	565 (67.6)	251 (54.9)	816 (63.1)
Both	53 (6.3)	12 (2.6)	65 (5.0)
Procedural VMMC provider gender			
Male	461 (55.1)	277 (60.6)	738 (57.1)
Female	375 (44.9)	180 (39.4)	555 (42.9)
Wound check provider gender			
Male	341 (40.8)	219 (47.9)	560 (43.3)
Female	454 (54.3)	207 (45.3)	661 (51.1)

Data are presented as No. (%). Percentages may not add up to 100% due to missing data.

Abbreviation: VMMC, voluntary medical male circumcision.

### Satisfaction With Voluntary Medical Male Circumcision Experience

**Figure 1.**
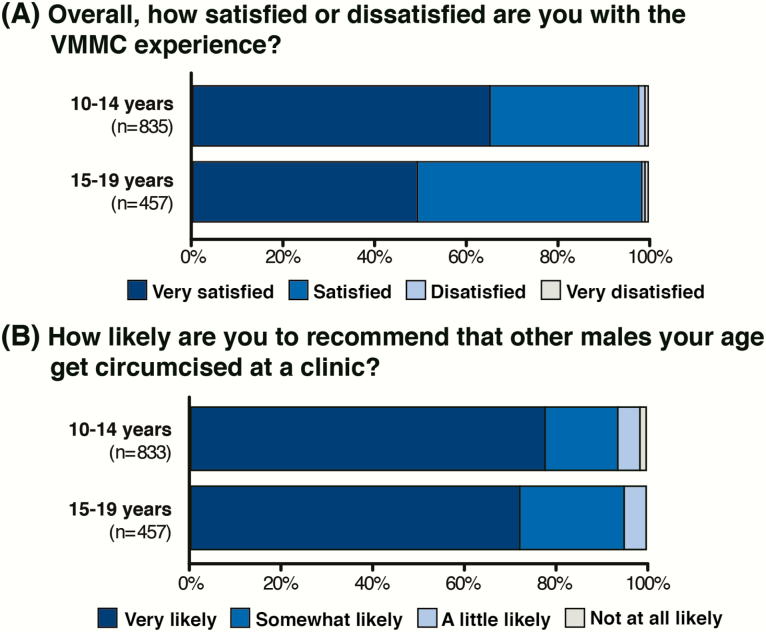
*A* and *B*, Overall satisfaction with the voluntary medical male circumcision (VMMC) experience.

The majority of adolescent clients (97.7% of 10- to 14-year-olds and 98.7% of 15- to 19-year-olds) reported they were either satisfied or very satisfied with their VMMC experience (Figure 1). When asked how likely they were to recommend VMMC to other males their age, 77.7% of 10- to 14-year-olds and 72.2% of 15- to 19-year-olds reported they were very likely to do so, with most of those remaining (15.9% and 22.5%, respectively) reporting they were somewhat likely to do so (Figure 1).

### Perceived Quality of In-service (Counselor) Communication

The overall perceived quality of in-service communication was high; the median perceived quality of in-service communication score was 8 (IQR, 7–9) among 10- to 14-year-olds and 9 (IQR, 8–9) among 15- to 19-year-olds (Supplementary Table 1). Age was independently associated with the perceived quality of in-service communication (score <7). Adolescents aged 10–14 years were more likely than 15- to 19-year-olds to perceive in-service communication to be of lower quality despite adjusting for country, facility setting, and receipt of postprocedure counseling session (aPR, 1.61 [95% CI, 1.33–1.95]; Table 2).

**Table 2.** Factors Associated With Low Perceived Quality of In-service (Counselor) Communication

Characteristic	% (n/N)	PR (95% CI)^ a ^	aPR (95% CI) ^ b ^
Age group, y			
10–14	21.5 (178/829)	**1.65 (1.29–2.10**)	**1.61 (1.33–1.95**)
15–19	8.2 (37/451)	Ref.	Ref.
Facility setting			
Rural	17.7 (71/402)	Ref.	Ref.
Periurban	14.1 (27/191)	1.02 (.27–3.77)	**0.50 (.37–.68**)
Urban	17.0 (117/687)	1.24 (.27–5.56)	1.17 (.82–1.67)
Preprocedure counseling			
Individual only	7.0 (23/327)	Ref.	…
Group only	12.8 (58/452)	1.31 (.84–2.04)	…
Both	26.3 (130/494)	1.20 (.55–2.60)	…
Preprocedure counselor gender			
Male only	9.5 (38/401)	Ref.	…
Female only	19.6 (158/808)	1.05 (.61–1.81)	…
Both	26.2 (17/65)	1.12 (.50–2.51)	…
Parent/guardian attendance at preprocedure counseling session			
No	9.9 (74/749)	Ref.	…
Yes	26.3 (138/525)	1.36 (.86–2.13)	…
Received postprocedure counseling session			
No	23.0 (202/878)	Ref.	Ref.
Yes	3.2 (13/402)	0.64 (.35–1.18)	0.57 (.20–1.66)

This analysis was conducted to identify correlates of was a low perceived quality of in-service (counselor) communication score (<7/9) since scores were generally high (median, 8 [interquartile range, 7–9]). Prevalence ratios for a score <7 were calculated by modified Poisson regression models with generalized estimating equations and robust variance estimators to account for clustering of responses at the facility level. Factors shown to have an association with the outcome after adjustment for country were included in the final multivariable model (*P* < .15), with the exception of age group and the receipt of secondary counseling after the procedure, which were included regardless of statistical significance; Effect sizes for country are not shown. Estimates in bold have a *P* value < .05.

Abbreviations: aPR, adjusted prevalence ratio; CI, confidence interval; PR, prevalence ratio.

^a^Unadjusted prevalence ratios for each covariate shown.

^b^Final multivariable model included adjustment for country, age, facility setting, and receipt of postprocedure counseling session.

Facility setting was independently associated with a lower perceived quality of in-service communication (Table 2). When comparing periurban and urban settings to rural settings, those in periurban settings were significantly less likely to perceive in-service communication as lower quality (aPR, 0.50 [95% CI, .37–.68]; Table 2). No significant difference was found between rural and urban facilities. The mode of preprocedure counseling, preprocedure counselor gender, parent/guardian’s attendance at the preprocedure counseling session, and receipt of a postprocedure counseling session did not appear to significantly affect the perceived quality of in-service communication.

These findings were replicated in a sensitivity analysis limited to participants who received only preprocedure counseling (ie, excluding individuals who also received a postprocedure counseling session) (Supplementary Table 2).

### Influence of Counselor and Provider Gender

**Figure 2.**
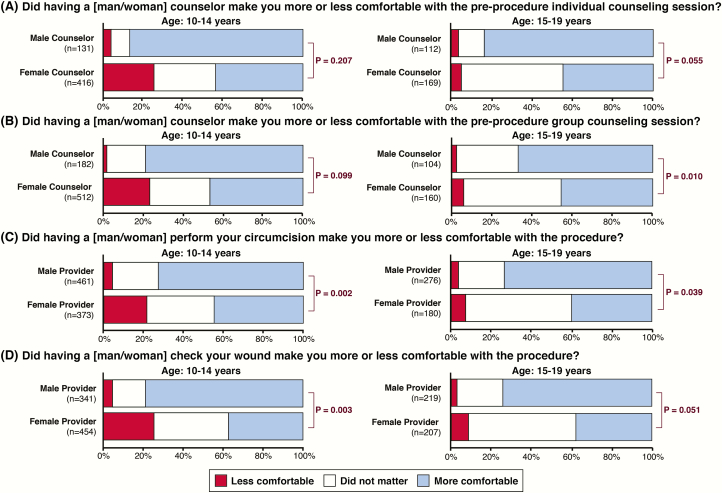
*A–D*, Gender preferences in the provision of voluntary medical male circumcision counseling and services among adolescents. Differences in the distribution of ordinal Likert responses between groups were assessed using the nonparametric Somers D test with Fisher Z transformation; the Somers D tests accounted for facility-level clustering using the delta-jackknife method and adjusted for country by stratification.

Adolescents in both age groups tended to feel more comfortable having a male rather than female counselor during both individual and group preprocedure counseling sessions, but this was only significant among 15- to 19-year-olds receiving group counseling after adjustment for country. In comparison to reported comfort among adolescents who had a female provider, both age groups were significantly more comfortable with a male provider performing the procedure and with a male provider being the one checking their wound during the follow-up appointment (Figure 2).

### Facility-Level Contributions to an Adolescent-Friendly Environment

**Figure 3.**
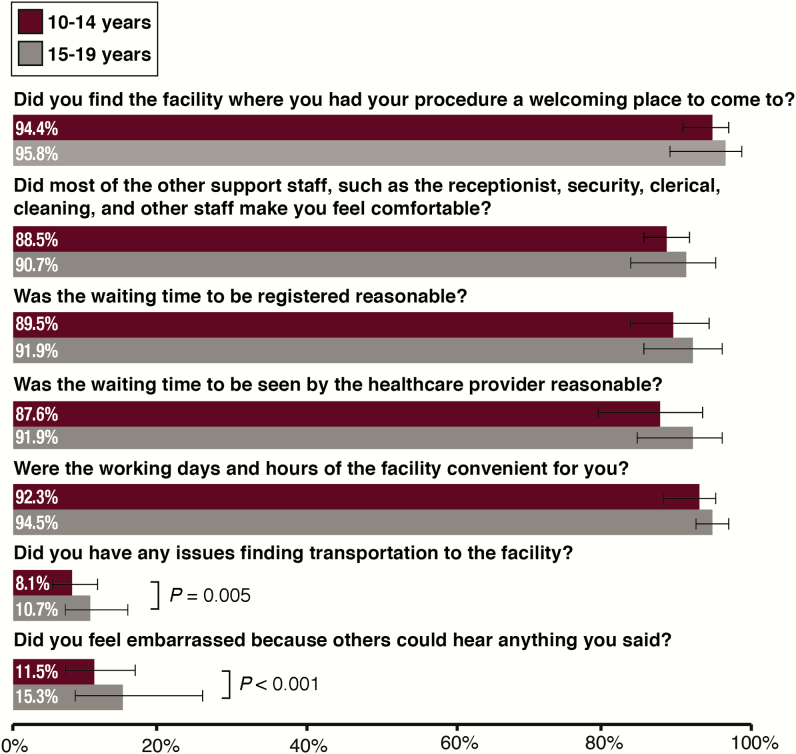
Adolescent perceptions of facility-level factors of service delivery. Proportions reflect a response of “yes.” 95% confidence intervals were estimated by Taylor series linearization. *P* values were calculated from modified Poisson regression models with generalized estimating equations and robust variance estimation; models were adjusted for country. *P* values >.05 are not shown.

Overall, adolescents had positive perceptions of the facilities where they received VMMC. The majority reported the facilities to be welcoming, they were made to feel comfortable by all staff, wait times were reasonable, and the hours of the facility were convenient (Figure 3). A significant difference was found between the 2 age groups when asked about finding transportation to the clinic and feeling embarrassed because others might overhear what was being said during their counseling session. The 10- to 14-year-olds were less likely to report issues with finding transportation than the older age group (8.1% vs 10.7%, respectively; *P* = .005) and were less likely to report feeling embarrassed because others might overhear what was said (11.5% vs 15.3%, respectively; *P* < .001). Although there were significant differences between the 2 groups (independent of country), the majority had not experienced transport issues or embarrassment during the counseling session.

### Adolescent Opinions on Counseling Topics

When asked what topics they wish had been discussed more during the counseling sessions, both 10- to 14-year-olds and 15- to 19-year-olds reported they wish there had been more discussion about pain (35.7% and 45.0%, respectively), followed by wound care (29.6% and 30.0%, respectively) and healing time (13.9% and 20.0%, respectively) (Table 3). Adolescent respondents reported they would like more information about partial human immunodeficiency virus (HIV) protection (13.0% for 10–14 years of age and 12.5% for 15–19 years of age), the VMMC procedure (11.7% for 15–19 years of age), side effects (13.3% among 15- to 19-year-olds), and condom use (12.2% among 10- to 14-year-olds). However, when asked what they wish had not been discussed, the same 3 topics—pain, wound care, and healing time—were most frequently mentioned (7.8%, 10.8%, and 11.0%, respectively, for both age groups combined) along with partial HIV protection and the VMMC procedure among 15- to 19-year-olds (7.4% for both).

**Table 3:** Topics Participants Wished Had Been Discussed More or Less During Counseling, by Age Group

Response	What Do You Wish Had Been Discussed (More) During Counseling?			What Do You Wish Had Not Been Discussed During Counseling?		
	10–14 y (n = 115)	15–19 y (n = 120)	Overall (N = 235)	10–14 y (n = 499)	15–19 y (n = 391)	Overall (N = 890)
VMMC						
Procedure	11 (9.6)	14 (11.7)	25 (10.6)	21 (4.2)	29 (7.4)	50 (5.6)
Side effects	10 (8.7)	16 (13.3)	26 (11.1)	15 (3.0)	8 (2.1)	23 (2.6)
Pain	41 (35.7)	54 (45.0)	95 (40.4)	41 (8.2)	28 (7.2)	69 (7.8)
Wound care	34 (29.6)	36 (30.0)	70 (29.8)	44 (8.8)	52 (13.3)	96 (10.8)
Healing time	16 (13.9)	24 (20.0)	40 (17.0)	48 (9.6)	50 (12.8)	98 (11.0)
Abstain from sex for 6 weeks	1 (0.9)	3 (2.5)	4 (1.7)	1 (0.2)	1 (0.3)	2 (0.2)
Abstain from masturbation for 6 weeks	1 (0.9)	2 (1.7)	3 (1.3)	0 (0.0)	3 (0.8)	3 (0.3)
HIV						
Partial HIV protection	15 (13.0)	15 (12.5)	30 (12.8)	27 (5.4)	29 (7.4)	56 (6.3)
HIV testing and counseling	8 (7.0)	5 (4.2)	13 (5.5)	3 (0.6)	4 (1.0)	7 (0.8)
HIV disclosure	1 (0.9)	4 (3.3)	5 (2.1)	1 (0.2)	3 (0.8)	4 (0.5)
HIV heterosexual transmission	3 (2.6)	5 (4.2)	8 (3.4)	2 (0.4)	2 (0.5)	4 (0.5)
HIV homosexual transmission	1 (0.9)	2 (1.7)	3 (1.3)	2 (0.4)	2 (0.5)	4 (0.5)
Varying risk by type of sex behavior	0 (0.0)	1 (0.8)	1 (0.4)	1 (0.2)	2 (0.5)	3 (0.3)
Non-HIV sex education						
Abstinence	0 (0.0)	2 (1.7)	2 (0.9)	4 (0.8)	6 (1.5)	10 (1.1)
Partner reduction	1 (0.9)	3 (2.5)	4 (1.7)	0 (0.0)	7 (1.8)	7 (0.8)
Condom use	14 (12.2)	11 (9.2)	25 (10.6)	14 (2.8)	8 (2.1)	22 (2.5)
Other STIs	2 (1.7)	2 (1.7)	4 (1.7)	0 (0.0)	1 (0.3)	1 (0.1)
Pregnancy prevention	3 (2.6)	2 (1.7)	5 (2.1)	2 (0.4)	3 (0.8)	5 (0.6)
General sexual health	0 (0.0)	4 (3.3)	4 (1.7)	1 (0.2)	1 (0.3)	2 (0.2)

Data are presented as no. (%).

Abbreviations: HIV, human immunodeficiency virus; STI, sexually transmitted infection; VMMC, voluntary medical male circumcision.

## DISCUSSION

Despite reporting high levels of satisfaction with their VMMC service and overall high perceived quality of in-service communication, younger adolescents were significantly more likely to perceive the in-service communication as lower quality compared to older adolescents. Overall, adolescents of all ages found facilities welcoming and reported feeling more comfortable with male counselors and providers compared with female counselors and providers. However, adolescents across all ages reported they would have liked to receive more information about pain, sexual health, wound care, and healing time, and the majority reported not receiving postoperative counseling or a combination of individual and group counseling, as recommended by WHO guidance.

The absence of a postprocedure counseling session is concerning since WHO guidance [16–18], the US President’s Emergency Plan for AIDS Relief (PEPFAR) external quality assurance assessment tools [19, 20], and country-specific guidelines [21, 22] require postoperative counseling to highlight the 6-week healing period and to include instructions on wound care and other key messages, including how to avoid risk of tetanus. The postprocedure counseling session aims to ensure client understanding and confirm follow-up appointments. Given that parents play an important role in supporting wound care and healing of their adolescent sons [23], if no postprocedure counseling session is performed, neither the adolescent client nor, by extension, his parents are likely to receive adequate information.

Furthermore, most adolescent clients, regardless of age, did not receive both group and individual preprocedure counseling sessions, although both are clearly defined in the WHO guidelines [16, 24]. The group education session is used to provide basic information about VMMC and should be followed by individual counseling to address an adolescent’s individual issues in a private and confidential setting. This information gap was demonstrated by the adolescents in this study through their desire for additional discussion on several topics related to VMMC, including the associated pain, which multiple studies have also shown is a primary concern for adolescent VMMC clients [25–28]. Without complete counseling, it is unlikely adolescents are receiving all of the information they need, which in turn increases their risk of postoperative complications and may influence their subsequent uptake of other services and quality care.

Beyond worries regarding pain, a main topic also reported by adolescents needing further discussion is sexual health. Younger adolescents aged 10–14 years, reported they would also like more information about condom use. As found in Kenya [29], providers may be hesitant and uncomfortable counseling younger adolescent clients on comprehensive sexuality education due to personal, cultural, and religious beliefs or because they assume that the adolescent is not yet sexually active or is too young to understand such issues [30]. These barriers infringe on the rights of adolescents to receive this much-needed information and present a missed opportunity to disseminate HIV prevention messages. It is essential to reach adolescents aged 10–14 years as their in-depth knowledge of sex and ways to prevent HIV acquisition, including the use of condoms, is often quite low; condom use among sexually active males in this age group is typically lower than that of older adolescents [31, 32]. As outlined in other findings from this study population [33], younger adolescents aged 10–14 years received fewer elements of the minimum package overall than did 15- to 19-year-olds, despite the fact that adolescents stated they wish they had more of this information.

This study has limitations. Given the cross-sectional study design, reported associations should be descriptively interpreted, and the measures for perceived quality must be taken in context of this particular study. The study population may contain selection biases, as the study only captured adolescents who underwent VMMC and completed the postprocedure survey. The responses of this study sample may vary from adolescents who did not complete the follow-up survey and adolescents who attended the service provision site but did not ultimately undergo VMMC.

While adolescents across the 3 study countries reported high perceived quality of in-service communication and would generally recommend VMMC to their peers, they also reported wanting additional discussion on topics including pain, wound healing, sexual health, and the VMMC procedure itself. As stated by the WHO, adolescents “are not simply older children or younger adults,” but instead are individuals with particular needs who require providers sufficiently competent to respond to those needs and ensure adolescent-responsive care [34]. VMMC programs are therefore expected to ensure that all counseling (group, individual, and a postprocedure session) is provided according to global guidance and all topics are discussed to the satisfaction of adolescent clients. When the specific needs of all adolescent clients are addressed through comprehensive counseling that considers the developmental competencies of individual clients [35], encompassing both their desires and the global guidelines, VMMC has the potential to have the greatest impact on both immediate and long-term male health-seeking behavior.

## Supplementary Data

Supplementary materials are available at *Clinical Infectious Diseases* online. Consisting of data provided by the authors to benefit the reader, the posted materials are not copyedited and are the sole responsibility of the authors, so questions or comments should be addressed to the corresponding author.

VanLith_Perceived_SupplementalFile_Final

## Notes

***Acknowledgments.*** We are grateful to the adolescent participants in this study for sharing their perspectives and experiences. The authors also thank the VMMC facility managers, community mobilizers, and providers for their support, and acknowledge the contributions of the Technical Advisory Group for the adolescent VMMC assessment and their guidance throughout the study. We are privileged to work with this group of dedicated professionals from the US President’s Emergency Plan for AIDS Relief (PEPFAR), Centers for Disease Control and Prevention, US Department of Defense, World Health Organization, United Nations Children’s Fund, and Bill & Melinda Gates Foundation. Thanks go to the Human Sciences Research Council in South Africa, CSK Research Solutions in Tanzania, PSI/Zimbabwe, and the Centre for Sexual Health and HIV/AIDS Research in Zimbabwe for their assistance with data collection. The authors also appreciate the assistance of Meaghen Murphy with copyediting and Maria Elena Figueroa with project startup.

***Financial support.*** This work was supported by USAID (cooperative agreement number AID-OAA-A-12-00058) to the Johns Hopkins Center for Communication Programs and co-funded by the UK Department of International Development through the Integrated Support Program in Zimbabwe.

***Supplement sponsorship.*** This article appears as part of the supplement “Adolescent Voluntary Medical Male Circumcision: Vital Intervention Yet Improvements Needed,” sponsored by Johns Hopkins University.

***Potential conflicts of interest.*** All authors: No reported conflicts of interest. All authors have submitted the ICMJE Form for Disclosure of Potential Conflicts of Interest. Conflicts that the editors consider relevant to the content of the manuscript have been disclosed.
