# Supplementary material for: Perceived Quality of In-Service Communication and Counseling Among Adolescents Undergoing Voluntary Medical Male Circumcision
Source: Clin Infect Dis. 2018 Apr 3;66(Suppl 3):S205–12. doi: 10.1093/cid/cix971 (PMC5888942; doi:10.1093/cid/cix971)
Supplement: VanLith_Perceived_SupplementalFile_Final [file cix971_suppl_vanlith_perceived_supplementalfile_final.pdf]

SUPPLEMENTAL INFORMATION

Supplemental Table 1. Psychometric properties of the perceived quality of in-service (counselor) communication scale.

**In-service (Counselor) Communication Scale** [*Theoretical Range* (0-9)]	**Loading**	**Overall**	**10-14 years**	**15-19 years**
Did the counselor give you his/her name and title?	0.6197	62.4% (804/1288)	57.3% (477/833)	71.9% (327/455)
Did the counselor greet you and ask your name?	0.5509	88.6% (1140/1287)	86.4% (720/832)	92.3% (420/455)
Did the counselor speak in a way you were able to understand?	0.8109	93.6% (1207/1289)	91.3% (761/834)	98.0% (446/455)
Did the counselor give his/her full attention to you (or the group) during the counseling?	0.8371	93.7% (1209/1290)	92.1% (769/835)	96.7% (440/455)
Did the counselor treat you in a way that made you feel respected?	0.7238	95.3% (1229/1290)	93.7% (782/835)	98.2% (447/455)
Did the counselor ask if you had questions about what he/she explained?	0.7730	76.2% (981/1288)	71.6% (597/834)	84.6% (384/454)
Did the counselor make you feel comfortable/at ease to get circumcised?	0.6650	95.1% (1227/1290)	94.1% (786/835)	96.9% (441/455)
Did the counselor answer your questions in a relaxed manner without seeming rushed or hurried?	0.7366	84.1% (1081/1286)	80.4% (670/833)	90.7% (411/453)
Did the counselor give you enough time to ask everything you wanted to ask?	0.7750	83.9% (1079/1286)	80.3% (669/833)	90.5% (410/453)
Observed Scale Median (interquartile range)		8 (7-9)	8 (7-9)	9 (8-9)
Observed Scale Mean (standard deviation)		7.7 (1.7)	7.5 (1.9)	8.2 (1.2)

Binary responses were coded as 0 “no/don’t remember” vs. 1 “yes”. Principal components analysis was conducted using a tetrachoric correlation matrix. Screeplot visualization and parallel analysis (1000 repetitions) suggested extraction of one factor, which explained 57.6% of the variance. The final 9-point scale was constructed from complete-cases derived from a sample size of 1280 participants (age 10-14 years, n=829; age 15-19 years, n=451). The KR-20 for the overall scale was 0.7391.

SUPPLEMENTAL INFORMATION

Supplemental Table 2. Sensitivity analysis of factors associated with low perceived quality of in-service (counselor) communication score (<7/9) limited to participants who only received pre-procedure counseling.

Factor	% (n/N)	aPR (95% CI)[a]	aPR (95% CI)[b]
**Age group, y**			
10-14	26.0% (169/650)	**1.52 (1.18-2.27)**	**1.52 (1.23-1.98)**
15-19	14.5% (33/228)	Ref.	Ref.
**Facility setting**			
Rural	24.9% (108/433)	Ref.	Ref.
Periurban	14.5% (27/186)	**0.47 (0.37-0.59)**	**0.47 (0.39-0.58)**
Urban	25.9% (67/259)	0.98(0.77-1.24)	1.01 (0.82-1.23)
**Preprocedure counseling**			
Individual only	14.3% (20/140)	Ref.	-
Group only	18.5% (50/271)	1.44 (0.90-2.28)	-
Both	27.9% (129/462)	1.17 (0.68-1.99)	-
**Preprocedure counselor gender**			
Male only	16.3% (34/209)	Ref.	-
Female only	24.8% (150/604)	1.20 (0.78-1.84)	-
Both	28.3% (17/60)	1.31 (0.83-2.09)	-
**Parent/Guardian attendance at pre-procedure counseling session**			
No	16.9% (66/390)	Ref.	
Yes	27.7% (134/484)	1.15 (0.83-1.60)	

This analysis excluded participants that received a secondary counseling session since the perceived quality of communication questionnaire did not specify which counselor was being assessed (see Supplemental Table 1). Adjusted prevalence ratios (aPR) of a score <7/9 were estimated by multivariable modified Poisson regression with generalized estimating equations and robust standard errors to account for clustering of responses at the facility-level. Effect estimates for country are not shown. Effect estimates in bold had a *P* value <0.05.

[a] Separate models for each covariate shown include adjustment for country.
[b] Multivariable model included adjustment for country, age group, and facility setting.
